# Has Lockdown and COVID-19 Led to a Change in the Characteristics of Deep Vein Thrombosis and Patients Who Are Afflicted With It?

**DOI:** 10.7759/cureus.32424

**Published:** 2022-12-12

**Authors:** Abbas Shahid, Nashma Aden, Hatim Alsusa, Adil Shaikh, Michelle Howard, Saravanan Narayanamoorthi, Taha Khan

**Affiliations:** 1 Vascular Surgery, Northern Care Alliance NHS (National Health Service) Foundation Trust, Manchester, GBR; 2 Manchester Medical School, University of Manchester, Manchester, GBR; 3 Stroke Medicine, Northern Care Alliance NHS (National Health Service) Foundation Trust, Manchester, GBR

**Keywords:** covid-associated vte, venous thromboembolism (vte), vte, thrombo embolic disease, general and vascular surgery, vascular, national lockdown, covid 19 impact of lockdown, covid 19, deep vein thrombosis (dvt)

## Abstract

Background: There is growing evidence identifying coronavirus disease 2019 (COVID-19) as a significant risk factor for thrombosis in inpatients. However, it remains uncertain if patients in the community have been influenced during the COVID-19 pandemic and national lockdown. This study, across four centres in the United Kingdom (UK), reviewed outpatients with deep vein thrombosis (DVT).

Aim: This study aims to find out whether lockdown and COVID-19 led to a change in the characteristics of DVT and patients who are afflicted with it, alongside a review of DVT service.

Methods: Data was collected retrospectively from electronic patient records system for the following periods: April 1 to June 30, 2019, and April 1 to June 30, 2020. These were the key months during the first national lockdown in UK. Data were analysed for patient demographics, risk factors, characteristics of DVT, management, and DVT reoccurrence. Statistical analyses were performed using GraphPad Prism 8 (Dotmatics, Boston, Massachusetts, United States).

Results: During the study periods, 227 outpatients from the community sustained DVT in 2019 and 211 in 2020. Of these patients, 13 in 2020 were COVID-19 positive. There was a difference in gender distribution with 128 males and 99 females in 2019, and 93 males and 118 females in 2020 (p= 0.0128). No significant difference was noted in the incidence of thrombophilia with nine in 2019 and three in 2020 (p=0.1437). Fewer long-haul journeys were made in 2020 (only two), compared to 16 in 2019 (p=0.012). Fewer patients had immobility as a risk factor in 2020 (n=55) compared to 2019 (n=79) (p=0.0494). However, there were more patients using oral contraceptive pills, with one in 2019 and nine in 2020 (p=0.0086) .

Conclusion: There is no significant difference in the characteristics, extent, and management of DVT prior to and during the COVID-19 lockdown. National lockdowns do not affect DVT in the community; however, it is important to highlight the surrounding inpatient numbers.

## Introduction

The coronavirus disease 2019 (COVID-19) pandemic drove the United Kingdom (UK) into three lockdowns; The first lockdown occurred from March 26 to July 4, 2020. During this lockdown, the general population was ordered to stay home from work and school, and all ‘non-essential’ establishments were closed [1], including restaurants and gyms. The second lockdown was implemented on November 5, 2020, and was lifted on December 2, 2020 [2]. The UK entered the third lockdown on January 5, 2021, which was lifted on March 8, 2021. During the second and third lockdowns, schools remained open [3].

As a result of these lockdowns, the general population may have been less active. A significant risk factor of venous thromboembolism (VTE) is immobilisation [4], which begs the question, to what extent, if any, have the national lockdowns affected the incidence of VTE in patients recovering from COVID-19 in the community?

On December 1, 2022, the World Health Organisation estimated a total of 24,000,101 COVID-19 infections in the UK, with 196,821 confirmed deaths [5]. Of those infected with COVID-19, 993,745 have been hospitalised in the UK during this pandemic [6], which leaves approximately 22,809,535 individuals recovering from COVID-19 in the community. Those infected with COVID-19 commonly present with symptoms such as a fever, shortness of breath, cough, and fatigue. Less common symptoms include diarrhoea, headache, and haemoptysis [7].

Furthermore, an increased incidence of VTE has been reported in those patients who have been hospitalised due to a COVID-19 infection [8]. Specifically, the incidence of pulmonary embolisms (PE) and deep vein thrombosis (DVT) have been markedly more prevalent, approximately 20%, in those with COVID-19 as opposed to patients who have been admitted to hospital for other conditions [9]. Furthermore, the rate of PE is increased in patients with acute respiratory distress syndrome (ARDS) due to COVID-19, as opposed to those with ARDS due to conditions unrelated to COVID-19 [10].

It is hypothesised that an inflammatory process triggered by infection with COVID-19 leads to a hyper-coagulable state [11], and many different mechanisms have been proposed to explain this. Firstly, COVID-19 has been shown to affect the angiotensin-converting enzyme receptor 2 (ACE2), which is present in pulmonary vessels. This results in the release of pro-inflammatory cytokines, such as interleukin 6 (IL-6), and chemokines [12]. These pro-inflammatory proteins then go on to cause epithelial damage and dysfunction, which leads to the activation of the coagulation cascade, as well as the complement pathway. Studies have shown that the aforementioned inflammatory cytokines, in particular IL-6, can be elevated in patients affected by COVID-19 [13,14]. Additionally, the action of COVID-19 on ACE2 results in the disruption of the renin-angiotensin pathway [15]. Anti-phospholipid antibodies have been reported in COVID-19 patients, which could possibly result in an increased risk of thrombosis [16]. However, this needs further exploration.

It has been recognised that patients with COVID-19 in hospitals have higher rates of VTE; however, the rates of VTE in the community have not been thoroughly investigated. The aim of this study is to determine whether the lockdown has influenced the characteristics of patients presenting with a DVT.

This article was presented as a poster at the International Society of Thrombosis and Haemostasis Virtual Congress hosted on July 17-21, 2021 in Philadelphia, and presented orally and as a poster at the Association of Surgeons in Training Annual Conference from March 4-6, 2022, in Aberdeen.

## Materials and methods

Study design

This study examined an observational cohort to assess if the implementation of national lockdown during the COVID-19 pandemic affected the characteristics and frequency of DVT in the community. The study was conducted across four secondary care centres serving a population distribution of approximately 800,000 people in the North West of England. Patient private information was anonymised for the purposes of this study.

Inclusion and exclusion

The inclusion criteria were: patients must have presented from the community, aged 18 or above, and must have had their DVT confirmed by a venous duplex scan. The exclusion criteria were hospital patients, those under the age of 18, and if no venous duplex had been conducted. All patients that met these inclusion criteria had their details stored in a secure database on the hospital server. We chose to only include patients that presented in the most restrictive lockdown in 2020 and then we compared this against the same time period in 2019. Patients included in the study presented to the service between April 1 to June 31, 2019, and April 1 to June 31, 2020. In 2019, 227 patients presented with a DVT compared to 211 in 2020.

Data collection

Data was collected for each respective patient using electronic records. Basic demographics were collected for each patient including age at the time of DVT diagnosis, gender, weight and body mass index (BMI).

This study compared patient characteristics between the 2019 and 2020 groups. These were listed as follows: smoking status, previous long bone fracture, inflammatory bowel disease, long haul travel, immobility, oral contraceptive pill (OCP)/hormone replacement therapy (HRT), pregnancy status, previous DVT, current or previous neoplastic disease, current PE, and COVID-19 status confirmed by polymerase chain reaction (PCR) test on presentation. Patients were each categorised as sedentary or active depending on their lifestyles and thrombophilia status recorded if there was any disease present such as anti-phospholipid syndrome, protein C or S deficiency, and factor V Leiden deficiency. If a patient had a previous DVT diagnosis, we attempted to characterise this into calf, popliteal, femoral, iliac, inferior vena cava or unknown site.

Outcome variables recorded included the proximal extent of each DVT, if they also had a concurrent contralateral DVT, its extent, and the anticoagulation treatment offered. Any other management and complications such as mortality, PE, or bleeding were also recorded.

Statistical tests

In order to analyse and compare the 2019 and 2020 data sets, a mixture of statistical tests was used depending on the variable subtype. For continuous variables such as age, weight, and BMI, we use a paired T-test. Dichotomous data was placed in contingency tables and a two-sided Fischer’s exact test was performed. Lastly, for categorical variables such as the extent of DVT, the chi^2^ test was used. The significance level was set with a p-value of less than 0.05 for all tests. To conduct all statistical analyses and formulate graphs and charts, the GraphPad Prism 8 software (Dotmatics, Boston, Massachusetts, United States) was used.

## Results

**Figure 1:**
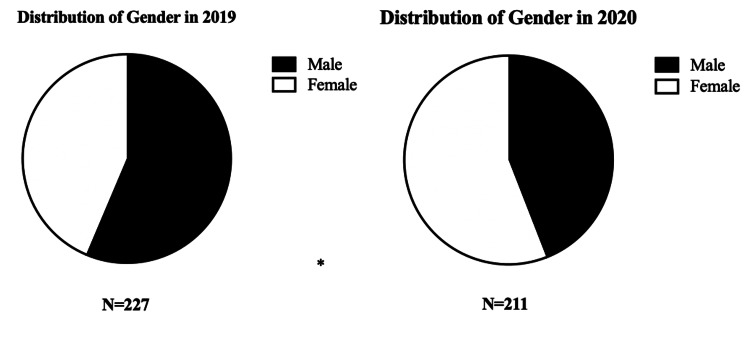
Comparing gender distribution between 2019 and 2020 *(p<0.05)

Analysis of the data did not reveal a significant difference in the majority of characteristics in 2020 compared to 2019 (Table 1). The 2019 cohort did not have the presence of the exposures observed with the 13 COVID-19-positive patients in the 2020 cohort and the enforcement of a national lockdown restricting movement. However, there was a statistically significant difference (p= 0.0128) in the distribution of genders as shown in Figure 1 with 128 males and 99 females in the 2019 observed cohort compared to 93 males and 118 females in the 2020 COVID-19 lockdown cohort. Furthermore, no significant difference was found in the number of people developing DVT, with 227 in the 2019 cohort and 211 in the 2020 cohort. 

**Table 1:** Summary of results of risk factors and DVT characteristics for each variable of results NS: not statistically significant; BMI: body mass index; IBD: inflammatory bowel disease; DVT: deep vein thrombosis; OCP: oral contraceptive pill; HRT: hormone replacement therapy; COVID-19: coronavirus disease 2019; PE: pulmonary embolism

		2019	2020	p-value
Age at DVT diagnosis		62.29±17.93	59.88±18.48	NS
Gender	Male	128	93	≤ 0.05
	Female	99	118	
Weight		82.84±20.14	81.94±19.88	NS
BMI		28.69±6.085	28.68±5.61	NS
Smoking	Yes	103	103	NS
	No	124	108	
Long bone fracture	Yes	14	23	NS
	No	213	188	
IBD	Yes	2	4	NS
	No	225	207	
Long haul travel	Yes	16	2	≤ 0.01
	No	211	209	
Immobility	Yes	79	55	≤ 0.05
	No	148	156	
OCP/HRT	Yes	1	9	≤ 0.01
	No	226	202	
Occupation	Sedentary	159	157	NS
	Active	68	54	
Pregnancy	Yes	5	9	NS
	No	222	202	
Thrombophilia	Yes	9	3	NS
	No	218	208	
COVID-19 Status	Positive	N/A	13	-
	Negative		198	
Previous DVT	Yes	105	86	NS
	No	122	125	
Types of Previous DVT	Calf	19	16	≤ 0.05
	Popliteal	12	6	
	Femoral	24	22	
	Iliac or above	16	3	
	Unknown	34	39	
Current Cancer	Yes	20	13	NS
	No	207	198	
Previous Cancer	Yes	17	17	NS
	No	210	194	
Current PE	Yes	12	13	NS
	No	215	198	
Wells score assessed	Yes	88	80	NS
	No	139	131	
DVT extent	Calf	77	64	NS
	Popliteal	31	22	
	Femoral	90	89	
	Iliac or above	29	20	
Contralateral DVT	Yes	11	8	NS
	No	216	202	
Extent of Contralateral DVT	Calf	4	2	NS
	Femoral and above	7	6	
Re-occurrence of DVT	Yes	16	22	NS
	No	211	189	
Anticoagulated	Yes	193	185	NS
	No	30	25	

**Figure 2:**
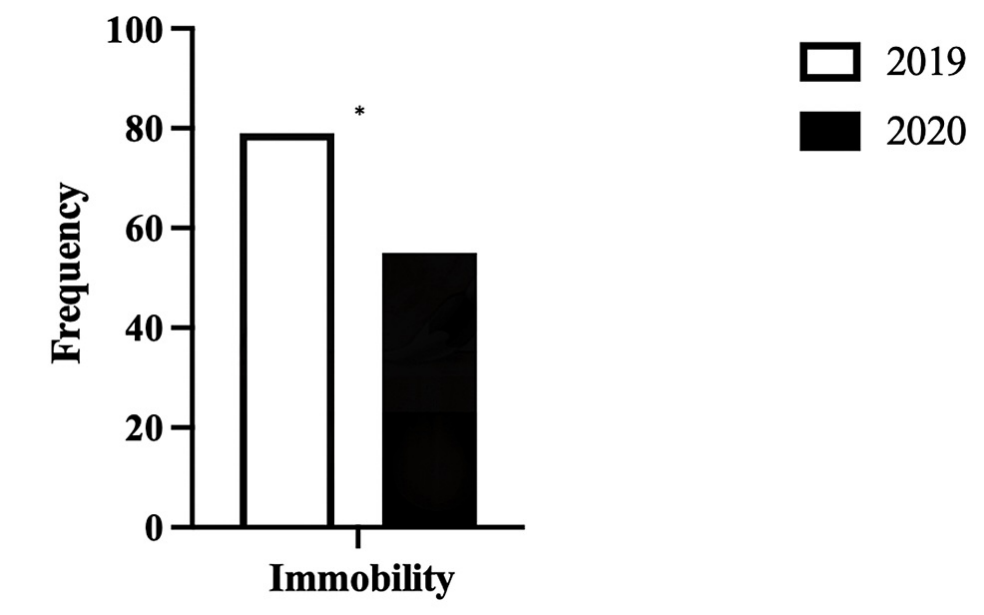
Comparison of functional immobility between the 2019 and 2020 cohorts *(p<0.05) DVT: deep vein thrombosis

There was a reduction in functional immobility observed as shown in Figure 2 between the two cohorts, with the 2020 cohort having a significantly lower proportion of people deemed to be immobile on their assessments. There were 79 people in 2019 as opposed to 55 in 2020 (p=0.0494). 

**Figure 3:**
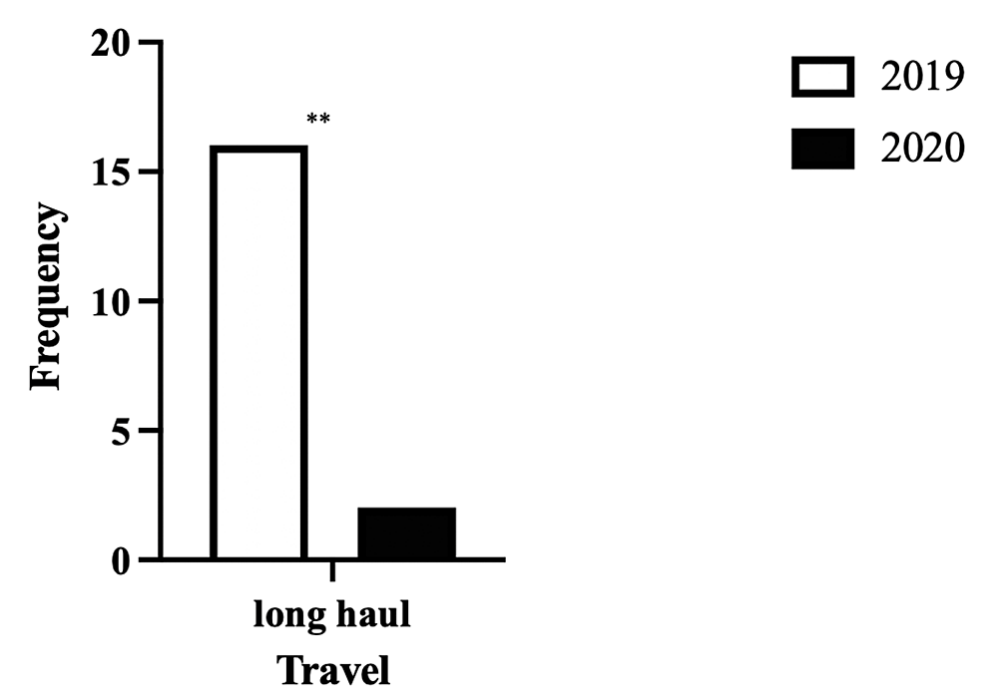
Comparison of the number of patients between 2019 and 2020 who went on long-haul travel ** (p<0.01)

As expected, given a national restriction in movement there were significantly fewer long-haul journeys made by the 2020 cohort compared to the 2019 cohort. As shown in Figure 3, there were 16 patients who had this risk factor as opposed to only two in 2020 (p=0.012).

**Figure 4:**
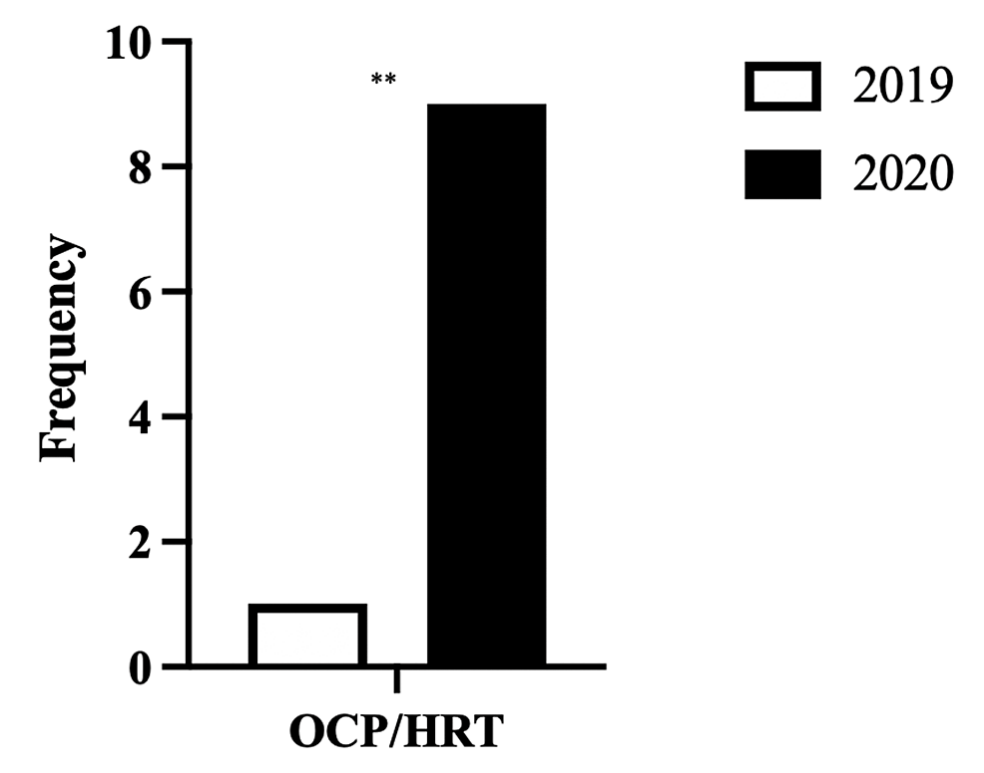
Comparison of the number of patients in 2019 and 2020 taking OCT/HRT ** (P<0.01) OCT: oral contraceptive pill; HRT: hormone replacement therapy

In the 2020 cohort, there was a greater prevalence (p=0.0086) of people using hormonal treatments such as the OCP and HRT. In 2019, there was only one person using OCP. However, eight people in 2020 were using OCP and one was using HRT (Figure 4). 

There was no significant difference in the incidence of thrombophilia with nine in 2019 and three in 2020 (p=0.1437). Similarly, as shown in Table 1, all other risk factors for VTE and characteristics of DVT had no statistically significant difference when comparing the 2019 and 2020 cohort groups.

Complications were observed in both cohorts. In 2019, there was a total of 16 deaths and two patients sustaining minor haemorrhages. In 2020, there were seven deaths recorded within six months of diagnosis and one patient developed a pulmonary embolism.

## Discussion

The national lockdown imposed in the UK has not made a significant difference in the frequency and characteristics of DVT in the community. The national lockdown was a necessary measure to thwart the spread of the COVID-19 virus, which was found to have a higher degree of mortality and virulence than standard severe acute respiratory syndrome (SARS) viruses [17]. A highly restrictive lockdown was essential to restrict movement, curb mortality, and protect limited healthcare resources such as ventilators and intensive care unit beds [18]. In secondary care, elective care was largely put on hold, and routine hospital appointments were postponed. Primary care changed to a telephone consultation system. Due to the airborne respiratory transmission of droplets, the virus was and still is highly infectious, and limiting movement and social contact was essential to decrease the rate of transmission in order to control infection rates.

Although patients in the community were not observed to be affected from a VTE perspective, they were significantly affected throughout the COVID-19 global pandemic in a multitude of ways. Socially, people suffered from limited interaction and isolation within their own homes [19]. Some suffered financially due to loss of income and social deprivation from losing work [20]. Patients were, in some cases, reluctant to get into contact with their general practitioner unless it was critically urgent to avoid unduly exposing themselves and their relatives.

This study demonstrated a statistically significant reduction in functional immobility in the 2020 cohort compared to the 2019 cohort (Figure 2). This was contrary to what was expected. Due to the national lockdown and restriction on movement, there was the expectation that there would actually be an increase in immobility from 2019 to 2020 as a result of this. A possible explanation for this finding is individuals were isolated within their homes due to guidelines and social distancing guidance and, as a result, a greater proportion of people involved themselves in physical activity in order to maintain general health and well-being [21,22].

Similarly, as shown in Figure 3, there was a significant reduction in long-haul travel from 2019 to 2020 as a risk factor for developing DVT. This can be explained by the implementation of the national lockdown as there were restrictions on the local movement of people as well as restrictions on travel between countries thereby reducing any opportunity for long-haul travel.

This study observed an increase in HRT use with a significant increase in OCP use (Figure 4), although studies report a reduction in sexual activity throughout the global pandemic [23]. This study identifies increased OCP use in patients with a DVT in 2020 when compared to the 2019 cohort. The 2020 cohort had a younger mean age compared to 2019 (Table 1); therefore, a larger proportion of the patient population may have been pre-menopausal and, therefore, eligible for OCP.

The limitations of this study include its retrospective nature and the inclusion only of patients presenting to the DVT service across the four large secondary care centres. A proportion of patients that may have been managed in the primary care setting will not have presented to the DVT service and therefore could not be included in this study. The study did not observe the long-term outcomes of patients with DVTs in the community and compared these across the two cohorts.

## Conclusions

There is no significant difference in the characteristics, extent, and management of DVT prior to and during the lockdown. National lockdowns do not affect community DVT characteristics in outpatients but COVID-19 has a significant impact on VTE in inpatients. Further large-scale epidemiological studies are needed to determine conclusively the impact of a lockdown on VTE in the community.
